# Editorial: Neurorobotics explores gait movement in the sporting community

**DOI:** 10.3389/fnbot.2023.1127994

**Published:** 2023-01-17

**Authors:** Andrej Gams, Ganesh R. Naik

**Affiliations:** ^1^Humanoid and Cognitive Robotics Lab, Department of Automatics, Biocybernetics and Robotics, Jožef Stefan Institute, Ljubljana, Slovenia; ^2^Adelaide Institute for Sleep Health, Flinders University, Bedford Park, SA, Australia

**Keywords:** exoskeleton, wearable robot, orthosis, interlimb neural coupling, human-exoskeleton interaction, hybrid BCI, locomotion

## Gait movement

Gait movement refers to the motion and pattern of how an individual walks. Gait movement is a complex combination of balance, movement (stance and swing phase), and coordination of different muscle groups. Neurological damage and disease can lead to impairments that prevent individuals from performing gait movement. Research within the Neurorobotics community has provided innovative solutions that can help reduce the time needed for rehabilitation.

This Research Topic aimed to compile research that focuses on gait analysis methods. In the wake of the successful Olympics and Paralympics in 2021, this Research Topic focused on research that should go beyond helping people with impairments, where the findings will apply also to the sporting community. We hope that the research uncovered will be able to provide cutting-edge technology for patients, able-bodied persons and athletes at the top of their game.

In the robotics and neurorobotics communities, gait is often studied through wearable robots—exoskeletons. The papers in this Research Topic all deal with such devices, their control, and utilization, but also focus on the rehabilitation aspects. The latter is not surprising—as stated by Lum et al. (2002), robot-assisted gait training reduces the working load of therapists and shortens the rehabilitation cycle for patients. While various mechanisms have been developed in the past, with extensive reviews available (Bao et al., 2019; Sanchez-Villamanan et al., 2019; Gull et al., 2020; Gupta et al., 2020), the mechanical design, control, and interface all remain open research challenges which have not been solved to the point of freely available and accessible wearable exoskeletons for everyday and clinical use. With this Research Topic, we aim to bring these things one step closer.

## Research Topic on gait movement

This Research Topic brings experimental findings and theoretical contributions to the field of gait science, tackled from the point of view of assistance with wearable devices, interfaces, and data processing for these tasks. It outlines guidelines for research activities in these field with concrete contributions in the development of methodologies and devices that fit in this Research Topic from the robotics perspective.

Different sub-topics are presented under the common Research Topic umbrella.

- Development of exoskeletons/orthosis and control, as discussed in Mu et al. and Zhang et al.. Exoskeletons have attracted increasing research interest in rehabilitation. The papers in this Research Topic provide a test-bed for future investigation of interlimb neural coupling Mu et al. and an evaluation of different assistive strategies on the interaction forces between the device and the wearer. Both of these aspects are informative for further development of mechanisms that can aid in rehabilitation (Han et al., 2022) and in efforts to assist able-bodied users (Fang and Hunt, 2021).
- Interaction through different modalities, as discussed by Zhang et al. and Tortora et al.. Interaction with the wearable device has explored different modalities: actual motion measured through the positions of the device or through inertial measurement units, interaction force and muscle activation as in electromyography (EMG), electroencephalography (EEG) as in brain-machine interface and hybrid combinations. The interface is often used to decode walking phases. The papers in this Research Topic deal with different modalities, showing how interaction forces can be assessed, how combinations of interface signals, i.e., hybrid human-machine interface (hHMI) outperforms single modalities and how different interfaces can be used to enhance the usability of technologies restoring or assisting the locomotion in a broader population of patients in clinical applications.
- Mode recognition as in Tortora et al. and Vu et al.. Locomotion mode recognition provides the wearable device control with information on when to switch between different walking modes. On the other hand, the gait phase detection indicates where one is in the gait cycle. However, these have to be determined based on the measured interface signals, such as the ones listed above. The recognition is essential for powered prostheses, which often implement a different control strategy for each locomotion mode to improve the functionality of the wearable device. Different methods of mode or action recognition have been employed in the past, but recently deep learning methodologies have started to dominate the field. The papers in this Research Topic show that different hybrid interface signals and different deep learning models were promising for applying locomotion mode recognition in real-time for robotic wearable devices and prostheses.

## Concluding remarks

Reading through the papers in the Neurorobotics explores Gait Movement in the Sporting Community Research Topic shows the different, interdisciplinary nature of robotics research on exoskeletons. Not only are mechanical design and biomechanics of the user in play, researchers tackle also interfaces, deep-learning and AI methodologies and work with users/patients in order to bring the field forward. The final goal, having viable and ready devices for everyday use in rehabilitation or in easing our daily activities, is near. The works in this Research Topic offer another stepping stone toward this goal.

The initial aim of this Research Topic was also in reaching toward the sporting community. The core messages of the papers offer insights that can help the sporting community. The main focus of exoskeletons currently remains with helping in rehabilitation and helping people with disabilities. Advancing the capabilities of able-bodied people remains a somewhat elusive goal.

## Author contributions

All authors listed have made a substantial, direct, and intellectual contribution to the work and approved it for publication.
